# Relaxin ligand/receptor systems in the developing teleost fish brain: Conserved features with mammals and a platform to address neuropeptide system functions

**DOI:** 10.3389/fnmol.2022.984524

**Published:** 2022-10-05

**Authors:** Anna Blasiak, Anna Gugula, Andrew L. Gundlach, Francisco E. Olucha-Bordonau, Francesco Aniello, Aldo Donizetti

**Affiliations:** ^1^Department of Neurophysiology and Chronobiology, Jagiellonian University, Krakow, Poland; ^2^Florey Department of Neuroscience and Mental Health, The Florey Institute of Neuroscience and Mental Health, The University of Melbourne, Parkville, VIC, Australia; ^3^Department of Anatomy and Physiology, The University of Melbourne, Parkville, VIC, Australia; ^4^UP Medicine, Faculty of Health Sciences, CIBERSAM, ISCIII, Universitat Jaume I, Castellón de la Plana, Spain; ^5^Department of Biology, University of Naples Federico II, Naples, Italy

**Keywords:** *griseum centrale*, neuropsychiatric disorders, *nucleus incertus*, relaxins, stress response, zebrafish

## Abstract

The relaxins (RLNs) are a group of peptide hormone/neuromodulators that can regulate a wide range of physiological processes ranging from reproduction to brain function. All the family members have originated from a RLN3-like ancestor *via* different rounds of whole genome and gene specific duplications during vertebrate evolution. In mammals, including human, the divergence of the different family members and the emergence of new members led to the acquisition of specific functions for the various relaxin family peptide and associated receptor genes. In particular, in mammals, it was shown, that the role of RLN3 is correlated to the modulation of arousal, stress responses, emotion, social recognition, and other brain functions, positioning this gene/peptide as a potential therapeutic target for neuropsychiatric disorders. This review highlights the evolutionary conservation of relaxin family peptide and receptor gene expression and their associated brain neural circuits. In the zebrafish, the expression pattern of the different relaxin family members has specific features that are conserved in higher species, including a likely similar functional role for the ancestral RLN3-like gene. The use of different model organisms, particularly the zebrafish, to explore the diversification and conservation of relaxin family ligands and receptor systems, provides a relatively high-throughput platform to identify their specific conserved or differential neuromodulatory roles in higher species including human.

## Overview of insulin-like/relaxin family peptides and receptors in mammals

Two relaxin genes (*RLN1* and *RLN2*) have been identified in humans (Crawford et al., 1984) and higher primates (Arroyo et al., 2014), whereas other mammals have only one gene (*Rln1*). Other insulin-like/relaxin family member genes were subsequently identified in humans (and other species): insulin-like peptide 3 (*INSL3*) (Adham et al., 1993); insulin-like peptide 4 (*INSL4*) (Koman et al., 1996); insulin-like peptide 5 (*INSL5*) (Conklin et al., 1999); insulin-like peptide 6 (*INSL6*) (Lok et al., 2000); and the most recently discovered, relaxin-3 (*RLN3*) (or insulin-like peptide 7, *INSL7*) (Bathgate et al., 2002; Burazin et al., 2002; Tanaka et al., 2005).

It is now widely accepted that RXFP1, RXFP2, RXFP3, and RXFP4 are the cognate receptors for RLN, INSL3, RLN3, and INSL5, respectively (Hsu et al., 2002; Hsu, 2003; Liu et al., 2003; Halls et al., 2007).

Relaxin peptide is considered a pleiotropic hormone acting as a paracrine, autocrine, and endocrine factor to mediate matrix remodeling, with numerous roles in and independent of reproduction, such as vasodilator and anti-fibrotic properties (Nistri et al., 2007; Du et al., 2010; Halls et al., 2015; Samuel et al., 2017). The numerous hormonal effects of relaxin are in accordance with the broad expression of its cognate receptor, RXFP1 (see Novak et al., 2006; Giordano et al., 2012; Bathgate et al., 2013; Chen et al., 2020 for details).

A clear role in male and female reproduction has been established for INSL3/RXFP2 signaling, since both *Insl3* and *Rxfp2* knockout male mice display a cryptorchid phenotype (Nef and Parada, 1999; Zimmermann et al., 1999; Overbeek et al., 2001; Gorlov et al., 2002), and an essential role in ovarian follicle maturation has been described (Pelusi et al., 2013; Ivell and Anand-Ivell, 2018).

The expression of *Insl5* and *Rxfp4* mRNA has been detected in the pancreas, thymus, eye and in the gastrointestinal tract (Conklin et al., 1999; Grosse et al., 2014), particularly in the L-cells of distal gut (Billing et al., 2018, 2019); and INSL5 has been shown to function as an orexigenic hormone (Grosse et al., 2014), with a role in control of colonic propulsion (Diwakarla et al., 2020).

RLN3 is considered a neuropeptide in mammals, since *Rln3*/*RLN3* mRNA/peptide is highly expressed in GABAergic neurons in the ventromedial pontine tegmentum, in an area known as the *nucleus incertus* (NI), and in the pontine raphe nucleus, the juxta-aqueductal ventral periaqueductal gray and the area adjacent to the substantia nigra in non-human primate, rat and mouse brain (Bathgate et al., 2002; Burazin et al., 2002; Liu et al., 2003; Tanaka et al., 2005; Ma et al., 2007, 2009; Smith et al., 2010). The cognate receptor for Rln3, Rxfp3 is widely expressed in many areas of the brain including the prefrontal and cingulate cortex, hippocampus, septum, thalamus, hypothalamus and the brainstem, and the pattern of RXFP3 expression largely overlaps NI efferent projections targets (Liu et al., 2003; Sutton et al., 2004; Smith et al., 2010). The broad expression of Rxfp3 in the brain is in line with suggested roles for Rln3/Rxfp3 signaling in modulation of stress responses, appetite, feeding and metabolism, motivation and reward, exploratory navigation, emotion (anxiety) and social recognition, memory and cognition, and sleep and circadian rhythm (Sutton et al., 2004; Gundlach et al., 2009; Smith et al., 2010; Tanaka, 2010; Callander et al., 2012; Ganella et al., 2013; Ma et al., 2013, 2017; Ryan et al., 2013; Kumar et al., 2017; de Ávila et al., 2018; Albert-Gasco et al., 2019; Kania et al., 2020).

There is little information available about the role of INSL4 and INSL6, and their target receptors are yet to be identified. Therefore, the following sections concentrate on the other relaxin family peptides, with a special focus on RLN3, a product of the ancestral relaxin family gene, as its expression and role in both fish and mammalian brain is well studied.

## Insulin-like/relaxin family peptide and receptor systems in teleost fish and mammals

Based on sequence conservation, a RLN3-like gene emerged as the ancestral member of the relaxin peptide family (Wilkinson et al., 2005a,b). This hypothesis was later corroborated by studies of gene expression patterns in the developing zebrafish (Donizetti et al., 2008, 2009), that revealed a pattern of mRNA localization that closely resembled that of mammalian *Rln3* mRNA in the mature brain of multiple species and developing mouse brain (Miyamoto et al., 2008). Subsequent phylogenetic, molecular evolutionary, and syntenic analyses established that teleosts contain orthologs of four relaxin family peptides (Good-Avila et al., 2009). A tripartite origin was proposed for the relaxin ligand/receptor system, whereby all vertebrate family members appear to have arisen from three genes in the ancestral vertebrate genome, one for the ligand (*ancrln*), and two for the receptors (*ancrxfp1/2* and *ancrxfp3/4*) (Yegorov et al., 2014). During vertebrate evolution, the repertoire of ligands and receptors increased differently between tetrapods and teleost fish, with significantly higher retention rates of *rxfp* genes in the latter (Yegorov and Good, 2012).

In humans and mice, the receptor to ligand ratio is close to one, although binding promiscuity accounts for the observations that ligands can bind to multiple receptors (Bathgate et al., 2013, 2018). The co-evolution of *rln*/*insl*/*rxfp* peptide and receptor genes in teleosts is different, with a receptor-ligand ratio in zebrafish higher than in tetrapod species (Good et al., 2012; Alnafea et al., 2019). In teleosts, there are multiple receptors for some ligands (for comprehensive analysis of relaxin ligand-receptor phylogenetics and coevolution see Good et al., 2012; Yegorov et al., 2014). Two duplicated *rln3* and *insl5* paralogs are present in zebrafish together with seven *rxfp3* paralogs (zebrafish lacks a *rxfp4* gene, but has an additional copy of the *rxfp3-3* gene, and *rxfp3-3a3*), while *rln* and *insl3* are in single copy together with one copy of *rxfp1* and three of *rxfp2* (Good et al., 2012; Yegorov et al., 2014). Retention of such multiple copies of ligand and receptor genes may be a consequence of the sub-functionalization process and provides the opportunity to dissect the role in specific territories/processes of the mammalian ortholog.

## Relaxin ligand/receptor system expression in the developing zebrafish

The study of the developmental expression pattern of relaxin family ligands and receptors in zebrafish provided some insights into their likely function in vertebrate embryogenesis, which is largely unexplored in mammals. Zebrafish possesses six genes for relaxin ligands and eleven for their putative receptors (Yegorov and Good, 2012). Most of them are expressed during embryonic development with many transcripts of maternal origin, and an expression level relatively higher at pharyngula and larval stages than early stages (Donizetti et al., 2008, 2009, 2010, 2015a,b; Fiengo et al., 2012, 2013; Venditti et al., 2018). The temporal expression pattern during zebrafish development offers an interesting insight into the evolution of duplicated genes. Regarding the paralog genes arising from the teleost-specific, third whole genome duplication, the zebrafish genome contains paralog genes for two relaxin family ligands, *rln3* and *insl5*, and three receptors, *rxfp2*, *rxfp3-2*, and *rxfp3-3*. All the paralog genes display a different expression pattern, highlighting the divergence of their regulatory regions, and corroborated by their spatial expression pattern (see below).

## Relaxin ligand/receptor system expression in the teleost fish brain

**FIGURE 1:**
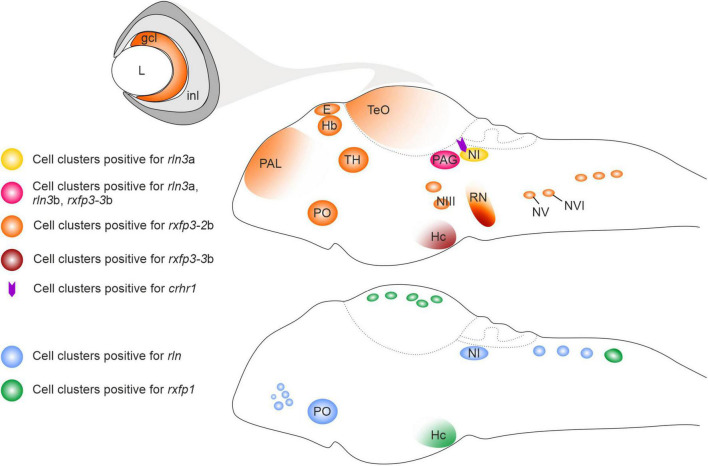
Schematic illustration of relaxin ligand and receptor expression patterns in the larval zebrafish brain. crhr1, corticotropin-releasing hormone type 1 receptor gene; ccl, ganglion cell layer; E, epiphysis; Hb, habenula; Hc, caudal hypothalamus; inl, inner cell layer; L, lens; NI, *nucleus incertus*; NIII, NIII cranial nerve nuclei; NV, NV cranial nerve nuclei; NVI, NVI cranial nerve nuclei; PAL, pallium; PAG, periaqueductal gray; PO, preoptic area; *rln*, relaxin; *rln*3a, relaxin peptide 3a gene; *rln*3b, relaxin peptide 3b gene; RN, raphe; *rxfp1*, relaxin family peptide type 1 receptor gene; *rxfp3*-2b, relaxin family peptide type 3 receptor 2b gene; *rxfp3*-3b, relaxin family peptide type 3 receptor 3b gene; TeO, optic tectum; TH, thalamus.

The distribution of *rln3*a expression during zebrafish development was in accordance with the embryonic expression pattern of the *Rln*3 gene during rat brain development (Miyamoto et al., 2008), with *rln3* mRNA-expressing cells located in two bilateral columns near the fourth ventricle, which likely correspond to the *nucleus incertus* (NI) (Donizetti et al., 2008). Despite this conservation, some interesting differences between zebrafish and rat were also observed. In the developing and adult rat brain, *Rln*3 transcripts are reported to be predominantly located in the NI, whereas a smaller number of *Rln3* mRNA-expressing neurons are present in other brain regions, such as periaqueductal gray (PAG), lateral substantia nigra and nucleus of the raphe pontis (Tanaka et al., 2005; Ma et al., 2007; Miyamoto et al., 2008; Blasiak et al., 2013). In zebrafish larva, *rln3*a gene expression appears first in the *griseum centrale* (GC), the homolog region of the mammalian PAG (Figure 1; Donizetti et al., 2008). The *rln3*b paralog gene is uniquely expressed in this territory during zebrafish brain development (Figure 1; Donizetti et al., 2009). Interestingly, the zebrafish *rln* gene, differently from the mammalian homolog *Rln1*, is expressed in the NI (Figure 1; Fiengo et al., 2012), probably as a consequence of an evolutionary conservation of regulatory elements for the expression in that territory associated with the ancestral gene. This expression pattern corroborated the hypothesis that the *rln* gene in fish is under different evolutionary pressures compared to the mammalian homolog gene, mimicking *rln3* gene (Good-Avila et al., 2009), and highlighting the putative relevance of the *rln3*-like ancestral gene function for the NI.

As predicted, relaxin family receptors display a wider expression in the developing zebrafish brain than their putative ligands (Figure 1; Donizetti et al., 2010, 2015a; Fiengo et al., 2013), highlighting an intricate network of functional connections between the different expression territories. At the larval stage, expression of *rxfp1* has been observed in different brain regions, among them the post-optic area, posterior tuberculum, hypothalamus, and cell clusters in the optic tectum and the rhombencephalic region (Donizetti et al., 2010). *Rxfp3-2*b is the receptor gene that displayed the widest expression, with cell clusters distributed in several brain regions, such as forebrain, pallium, epiphysis, habenula, thalamic regions and optic tectum, posterior tuberculum area of hypothalamus, medulla oblongata, and cranial nerve nuclei (Fiengo et al., 2013). *rxfp3-3*b transcripts were detected in the PAG, raphe and hypothalamus (caudal region) at relatively high levels, while weak hybridization signals were evident in the epiphysis, preoptic area, pons region and in the posterior rhombencephalic region (Figure 1; Donizetti et al., 2015a). Overall, the expression patterns of *rxfp3*-*2*b and –*3*b generally overlap the expression pattern of their mammalian ortholog (Liu et al., 2005; Sutton et al., 2004; Ma et al., 2007; Smith et al., 2010).

## Relaxins in the fish–functional implications

In zebrafish, *rln3*a is expressed in two neuron clusters belonging to the GC; and the *rln3*b gene is co-expressed with *rln3*a in one cell cluster, while *rln* is co-expressed with *rln3*a in the other cluster. The GC is already present in lampreys, the oldest currently living group of vertebrates, and the pattern of input and output connections of the GC/PAG is similar in lampreys, zebrafish and mammals (Olson et al., 2017). One of the major inputs to the GC in zebrafish, originates from the dorsal interpeduncular nucleus (dIPN), and the IPN is heavily innervated by the lateral part of the dorsal habenula (dHbL) (Okamoto et al., 2012). A very similar pattern of innervation is observed in mammals, where the medial habenula (mHb; homolog of dorsal habenula in fish) heavily innervates the IPN (Lima et al., 2017; Quina et al., 2017), and the PAG and the NI receive a strong innervation from the IPN (Goto et al., 2001; McLaughlin et al., 2017; Quina et al., 2017). Notably, the mHb-IPN pathway is one of the most evolutionarily preserved neural tracts in the forebrain from lamprey to human (Díaz et al., 2011; Stephenson-Jones et al., 2012; Villalón et al., 2012). The conservation of the GC/PAG region and its connectivity with other brain areas, suggests that a structure corresponding to the GC/PAG was present very early in vertebrate evolution and that the functions of the neuronal circuits associated with GC/PAG are similar in the vertebrate lineage.

**FIGURE 2:**
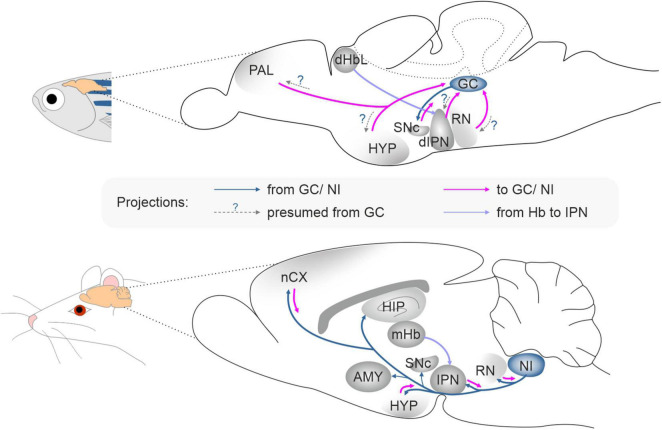
The connectivity pattern of the *griseum centrale* in zebrafish and the *nucleus incertus* in rat. AMY, amygdala; dHbL, dorsal habenula lateral part; dIPN, dorsal interpeduncular nucleus; GC, *griseum centrale*; HIP, hippocampus; HYP, hypothalamus; IPN, interpeduncular nucleus; nCX, neocortex; mHb, medial habenula; NI, *nucleus incertus*; PAL, pallium; RN, raphe nucleus; SNc, substantia nigra pars compacta.

In both fish and mammals, the GC/PAG and NI are embedded within neural circuits associated with aversion and stress-related behavior (Figure 2; Agetsuma et al., 2010; Okamoto et al., 2012; do Carmo Silva et al., 2018) and the Hb–IPN–GC/PAG/NI pathway has been suggested to control freezing and stress/aversion-related behaviors (Agetsuma et al., 2010; Yamaguchi et al., 2013). In rodents the RLN3/RXFP3 signaling system, with RLN3 neurons originating in the NI, has been implicated in the control of food intake, stress responses (including freezing), anxiety, addiction, locomotor activity, memory, and other arousal-related behaviors (Ma et al., 2013; Pereira et al., 2013; Blasiak et al., 2017; Kumar et al., 2017). Moreover, NI RLN3-synthetizing neurons have been shown to be directly sensitive to stress and arousal-related neurotransmitters, and to express corticotropin-releasing hormone type 1 receptors (CRHR1), orexin type 2 receptors (OX2), and melanin-concentrating hormone type 1 receptors (MCH1) (Blasiak et al., 2013, 2015; Ma et al., 2013, 2017; Kastman et al., 2016; Sabetghadam et al., 2018). Although these data relate to studies in rats, and the role of *rln3*a and *rln3*b expressing neurons in fish has not been verified, given the conservation of the relaxin family peptides and the predicted distribution of Rln3a and Rln3b peptides in corresponding, highly preserved structures in fish and mammalian brain (CG and PAG/NI, respectively), it is reasonable to consider that the role of these peptides is similar across these, and other vertebrate groups. Involvement of the RLN3/RXFP3 system in mammals and fish in stress-related behaviors is also supported by *crhr1* expression in NI neurons in zebrafish (Donizetti et al., 2008). These data suggest, that in fish as in mammals, the RLN3/RXFP3 system may be involved in control of behavioral activity levels, and specifically in CRH/CRHR1 system-induced hyperactivity (Donizetti et al., 2008; Ma et al., 2013; Lu et al., 2020; Faught and Vijayan, 2022). Finally, the expression of *rxfp3-2*b in areas of fish brain that broadly overlap the expression pattern of its mammalian ortholog, *RXFP*3 in rodent brain (Fiengo et al., 2013; Ma et al., 2017), reinforces the proposed parallel role for the *RLN3*/*RXFP3* system across vertebrates.

## Future directions

Relaxin family peptides and their receptors are crucial players in neural processes associated with survival and proper functioning in the environment, yet many questions regarding relaxinergic systems remain unanswered. The specific role and nature of relaxin family peptide signaling in structures such as amygdala, IPN, hippocampus and raphe nuclei, as well as the consequences of dysregulation of relaxin family peptides synthesis and their receptor functioning remain obscure and identify the need for expansion of current studies into new research areas using multiple techniques and tools. A high level of conservation of relaxin family peptides and their receptors, reflected by similarities in the structure of peptides and receptors, as well as their distribution in the brain in fish and mammals, makes the zebrafish an attractive experimental model for this field. Importantly, the very similar pattern of anatomical connections of structures shown to be part of the relaxinergic system in fish and mammals, is a strong predictor that these anatomical similarities are associated with functional commonalities in these groups of organisms. This particularly applies to the dHb/IPN/GC system, which in zebrafish was shown to be crucial for the control of learning, aversive behavior, directional-based decision making and social interactions (Agetsuma et al., 2010; Chou et al., 2016; Cherng et al., 2020; Palumbo et al., 2020); and is in line with data revealing that mammalian Hb/IPN/PAG/NI connections and interactions are involved in the control of the same processes (Ma et al., 2013; Pereira et al., 2013; Kumar et al., 2017).

Therefore, expanding research on relaxin peptides and their receptors in zebrafish provides a unique opportunity to answer further questions about their specific function in defined physiological processes, provide details of both anatomical and functional connections between relaxin peptide- and relaxin receptor-expressing brain structures, and determine the influence of relaxinergic system manipulations on animal behavior, including the involvement of relaxin family peptide and receptor systems in stress management. In this regard, zebrafish offers a powerful opportunity considering that the stress response system in fish is comparable to those of rodents and human. In fact, most stress-related genes show a high genetic homology between zebrafish and human/rodent analogs, together with similar neurochemical and neuroendocrine mechanisms related to physiological and behavioral stress responses (reviewed in de Abreu et al., 2021). In addition, the mammalian hypothalamic-pituitary-adrenal (HPA) axis, a well-known responder during stress, shares extensive structural and functional homologies with the hypothalamic-pituitary-interrenal (HPI) stress axis of zebrafish (Alsop and Vijayan, 2008, 2009a,b). In regard to stress research, a fruitful investigation would be the analysis of relaxin ligand/receptor gene expression under prenatal stress conditions. In particular, the zebrafish larva represents a valid complementary model to rodents (Steenbergen et al., 2011), whereby alterations in gene expression can be analyzed in relatively simple experimental paradigms (D’Agostino et al., 2019). However, one of the unresolved issues in zebrafish concerns the effective identification and localization of the various relaxin family peptides, to complement the anatomical expression of their corresponding mRNA species and provide a more complete picture of expression pattern regulation of these peptide/receptor systems. In addition, the profile of binding and activation of the receptors by key ligands also needs to be determined to gain information on the pairing relationships between ligands and receptors and to better elucidate the role of the different relaxins.

In terms of functional studies, the zebrafish offers a powerful platform to readily conduct loss-of-function studies and explore possible genetic compensation by paralog genes (reviewed in Salanga and Salanga, 2021). Currently, CRISPR/Cas9 technology represents a mature approach to generate gene knockout lines, and can be integrated with strategies to generate conditional KO lines (Hans et al., 2021), and it is potentially able to precisely label different alleles and follow the genotype of each allele and each cell (Li et al., 2019). A major advantage of using the zebrafish for functional studies of the relaxinergic system is the opportunity to focus on the larval stage when expression of the relaxin ligand/receptor genes is restricted to specific territories, but still largely reflects the expression in the adult zebrafish brain. Studies at this developmental stage are strategic considering that many methods can be applied in a high-throughput fashion, such as an automated system that can monitor and quantify multiple behavioral features in different experimental paradigms, including sleep (Tran and Prober, 2022) and drug effects (see e.g., Barros et al., 2008; Cornet et al., 2018). Therefore, these methods combined with genetic manipulations could accelerate the discovery of the roles of relaxinergic systems in zebrafish brain and help identify possible therapeutic approaches for clinical neuropsychiatric conditions.

## Author contributions

AB and AD proposed the topic of the manuscript. AB, AG, ALG, FA, and AD reviewed the literature. AB, AG, and AD wrote the original draft. ALG and FO-B critically edited and revised the manuscript. AB, AG, ALG, and AD conducted the final editing. All authors approved the submitted version.
